# Regulation of extracellular matrix elements and sarcomerogenesis in response to different periods of passive stretching in the soleus muscle of rats

**DOI:** 10.1038/s41598-018-27239-x

**Published:** 2018-06-13

**Authors:** Sabrina M. Peviani, Vinicius Guzzoni, Clara M. Pinheiro-Dardis, Yara P. da Silva, Alisson C. R. Fioravante, Adriana H. Sagawa, Gabriel B. Delfino, João L. Q. Durigan, Tania F. Salvini

**Affiliations:** 1 Department of Physical Therapy, São Carlos Federal University, São Carlos, São Paulo State Brazil; 2 Postdoctoral Fellowship, University of Brasília, Brasília, Federal District Brazil; 3 Graduate Program in Rehabilitation Sciences, University of Brasilia, Brasília, Federal District Brazil

## Abstract

Stretching is a common method used to prevent muscle shortening and improve limited mobility. However, the effect of different time periods on stretching-induced adaptation of the extracellular matrix and its regulatory elements have yet to be investigated. We aimed to evaluate the expression of fibrillar collagens, sarcomerogenesis, metalloproteinase (MMP) activity and gene expression of the extracellular matrix (ECM) regulators in the soleus (SOL) muscle of rats submitted to different stretching periods. The soleus muscles were submitted to 10 sets of passive stretching over 10 (St 10d) or 15 days (St 15d) (1 min per set, with 30 seconds’ rest between sets). Sarcomerogenesis, muscle cross-sectional area (CSA), and MMP activity and mRNA levels in collagen (type I, III and IV), connective tissue growth factor (CTGF), growth factor-beta (TGF-β), and lysyl oxidase (LOX) were analyzed. Passive stretching over both time periods mitigated COL-I deposition in the SOL muscle of rats. Paradoxically, 10 days of passive stretching induced COL-I and COL-III synthesis, with concomitant upregulation of TGF-β1 and CTGF at a transcriptional level. These responses may be associated with lower LOX mRNA levels in SOL muscles submitted to 10 passive stretching sessions. Moreover, sarcomerogenesis was observed after 15 days of stretching, suggesting that stretching-induced muscle adaptations are time-dependent responses.

## Introduction

Stretching is a common method used in sports activities and rehabilitation programs to prevent muscle shortening^1–4^ and improve limited mobility^5^. Skeletal muscle adaptations have been demonstrated through different pathways in response to passive stretching^6–9^. Potential mechanisms involved in stretching-induced skeletal muscle adaptations rely on preventing muscle atrophy^10–12^ and the deposition of new sarcomere units in rat muscles^13^. Furthermore, stretching frequency and duration seem to play a crucial role in skeletal muscle adaptations, as observed in our previous studies^8,14^. As such, differences in the stretching methods applied (continuous vs. intermittent) affect the muscle growth^6^ and viscoelastic properties of tendon structures^15^. In addition, immobilization approaches^16–18^ and stretching techniques have been investigated in denervated rat muscle^19^. However, there is limited evidence regarding the effect of different periods of stretching on ECM homeostasis.

Connective tissue consists mainly of ECM proteins, including collagens, and plays an important role in force transmission from muscle fibers^20^ and protection against contractile damage^21^. Type I (COL-I) and type III fibrillary collagens (COL-III) are the major structural protein in the skeletal muscle ECM^21^, while the basement membranes consist primarily of type IV collagen (COL-IV) networks^20^. Extracellular matrix degradation is an enzymatic process regulated by MMP activity and endogenous tissue inhibitors of metalloproteinases (TIMPs)^22^, while MMP-2 and MMP-9 are gelatinases expressed in skeletal muscle that degrade collagens, fibronectin, proteoglycan and laminin^23^. Transforming growth factor-beta (TGF-β) is a crucial cytokine responsible for producing COL-I, fibronectin, and connective tissue growth factor (CTGF)^24,25^, in addition to suppressing MMPs^20^. In fact, elevated TGF-β prompts a fibrotic response^26,27^. Additionally, lysyl oxidase (LOX) has been associated with ECM development and plays a critical role in fibrous collagen stabilization through the formation of covalent cross-links^28^. Indeed, collagen cross-linking is important in producing force during passive stretching^29^.

Immobilization induces muscle atrophy and the proliferation of connective tissue, caused by changes in collagen synthesis and degradation in muscle tissue^30,31^. While previous research has shown that muscle stretching minimizes connective tissue accumulation^1,32^, results regarding the effect of stretching on connective tissue deposition and ECM remodeling remain controversial^33^. For example, Zotz *et al*.^34^ found that passive stretching (3 times a week for 1 week) increased type-I collagen (COL-I) content and simultaneously reduced both type-III collagen (COL-III) and TGFβ1 levels in the soleus muscle of female aged rats. Moreover, Faturi *et al*.^18^ observed increased connective tissue content in the denervated SOL muscles of rats in response to 7 intermittent stretching sessions. In our study, both gene expression and MMP-2 activity rose in response to denervated SOL muscle stretching in rats^14^, although a single stretching session provoked no changes in MMP-2 and MMP-9 activity^9^. However, the effect of different time periods on stretching-induced ECM adaptations has not been investigated.

Thus, the aim of this study was to evaluate the expression of fibrillar collagens (COL-I, III and IV), sarcomerogenesis, MMP activity and gene expression of the main ECM regulators in the SOL muscle of rats submitted to different periods of passive stretching. We hypothesize that a large number of passive stretching sessions will reduce collagen content and synthesis, with a concomitant increase in MMP-2 activity and greater multiplication of sarcomeres (sarcomerogenesis) in the SOL muscle of rats.

## Results

### Sarcomerogenesis, MMP-2 activity and collagen expression in SOL muscles submitted to 10 or 15 days of passive stretching

**Figure 1:**
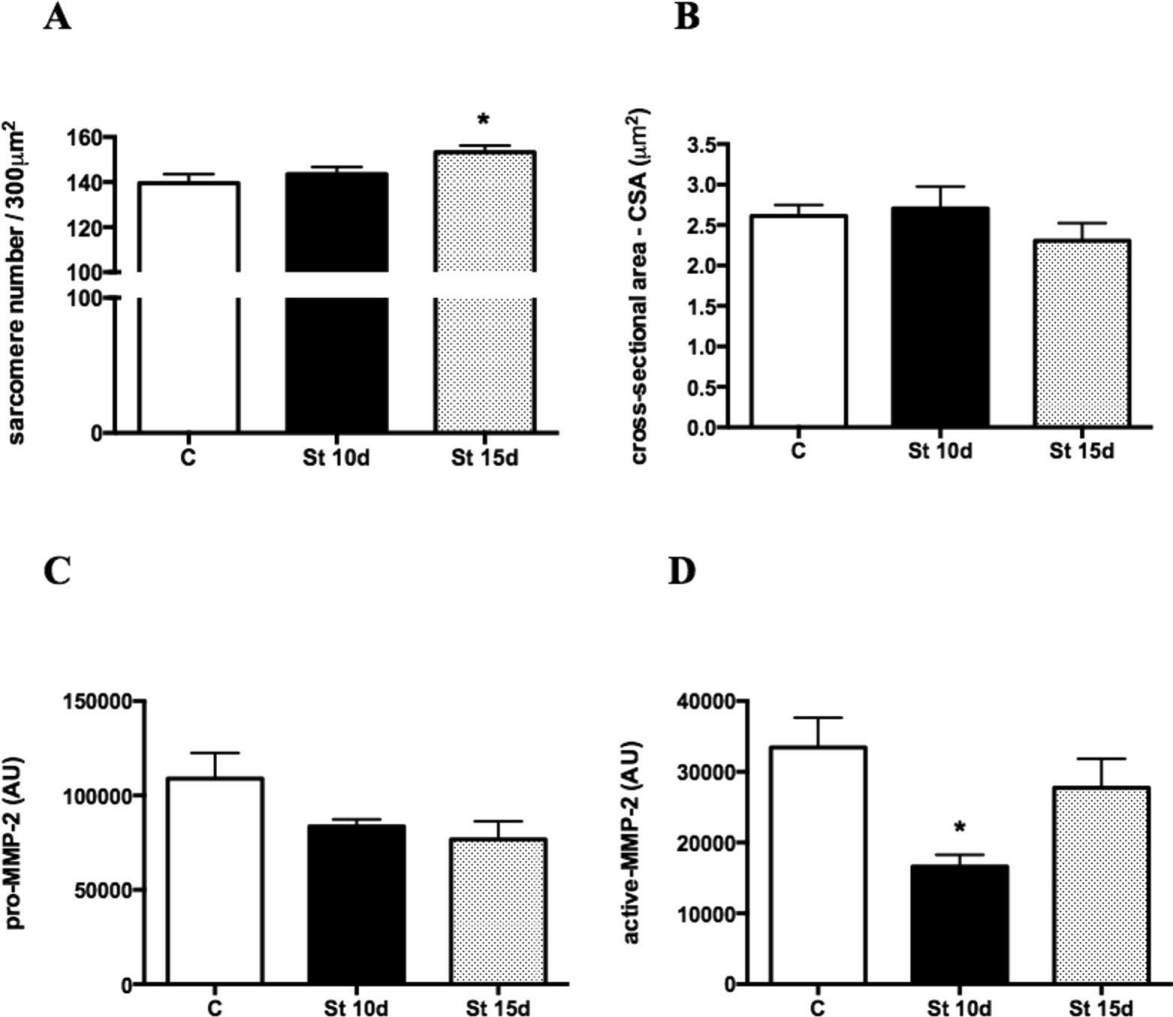
Sarcomere number (**A**), cross-sectional area - CSA (**B**), pro-MMP-2 levels (**C**) and MMP-2 activity (**D**) in SOL muscle. Groups: control (**C**), 10 days of passive stretching (St 10d) and 15 days of passive stretching (St 15d). Values are expressed as means ± SEM. One-way ANOVA, p < 0.05: * *vs*. C; ** *vs*. St 10d n = 6/group.

**Figure 2:**
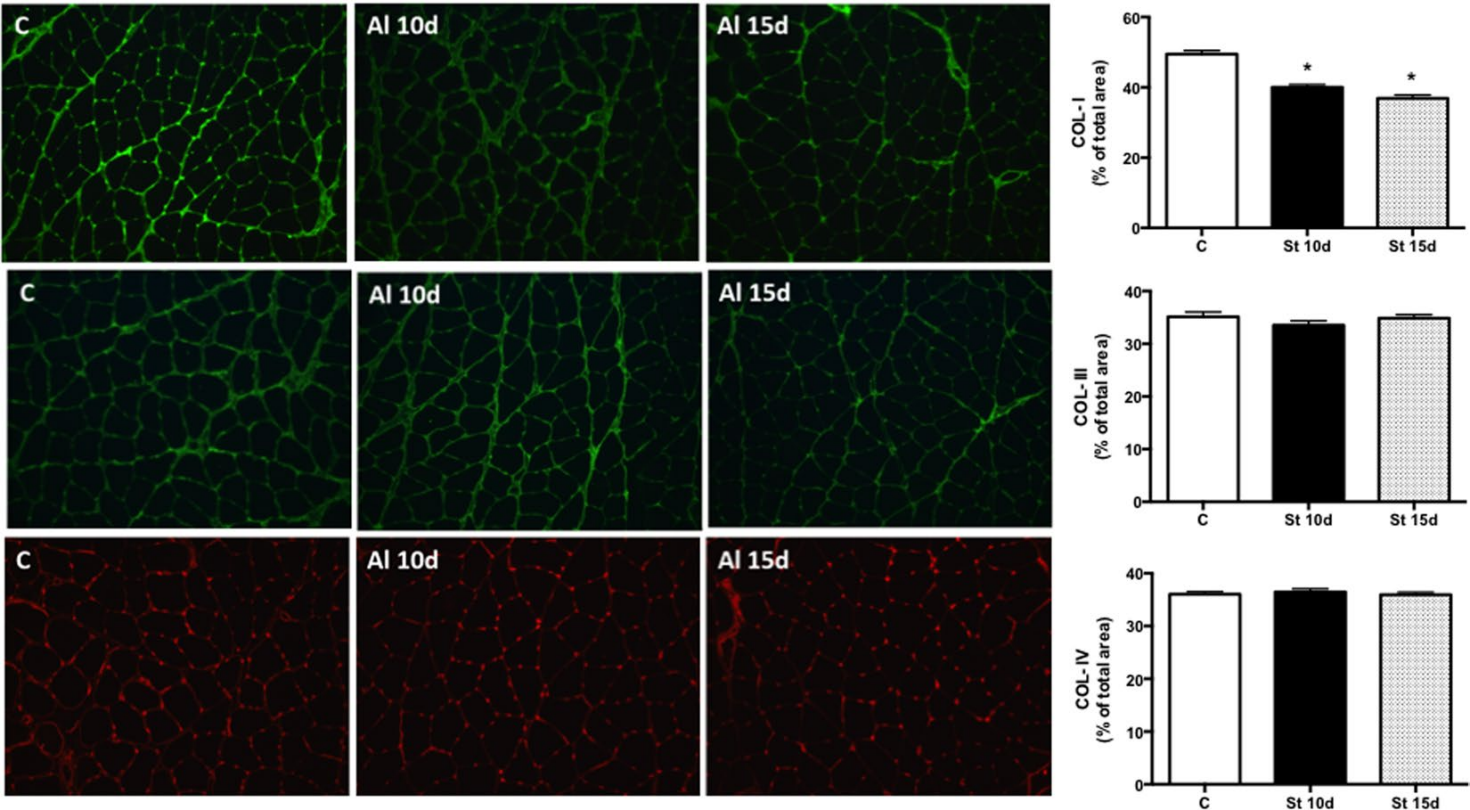
Representative images and quantification of COL-I (line A), COL-III (line B) and COL-IV (line C) levels in SOL muscle. Groups: control (C), 10 days of passive stretching (St 10d) and 15 days of passive stretching (St 15d). Values are expressed as % of area total ± SEM. One-way ANOVA, p < 0.05: * *vs*. C; ** *vs*. St 10d n = 6/group.

The sarcomere number in SOL muscles increased in the St 15d group when compared to controls (Fig. 1A; p < 0.05). There were no intergroup changes in CSA and pro-MMP-2 levels (Fig. 1B and C). MMP-2 activity declined in St 10d rats in relation to controls (Fig. 1D; p < 0.05). Figure 2 shows qualitative analysis of immunofluorescence staining for COL-I, III and IV. COL-I deposition (% per muscle fiber area) decreased in the SOL muscles of groups St 10d and St 15d when compared to controls (Fig. 2A; p < 0.0001). There were no intergroup differences in COL-III and COL-IV levels (Fig. 2B and C).

### mRNA levels of COL-I, III and IV, TGF-β1, CTGF and LOX in SOL muscles submitted to 10 or 15 days of passive stretching

**Figure 3:**
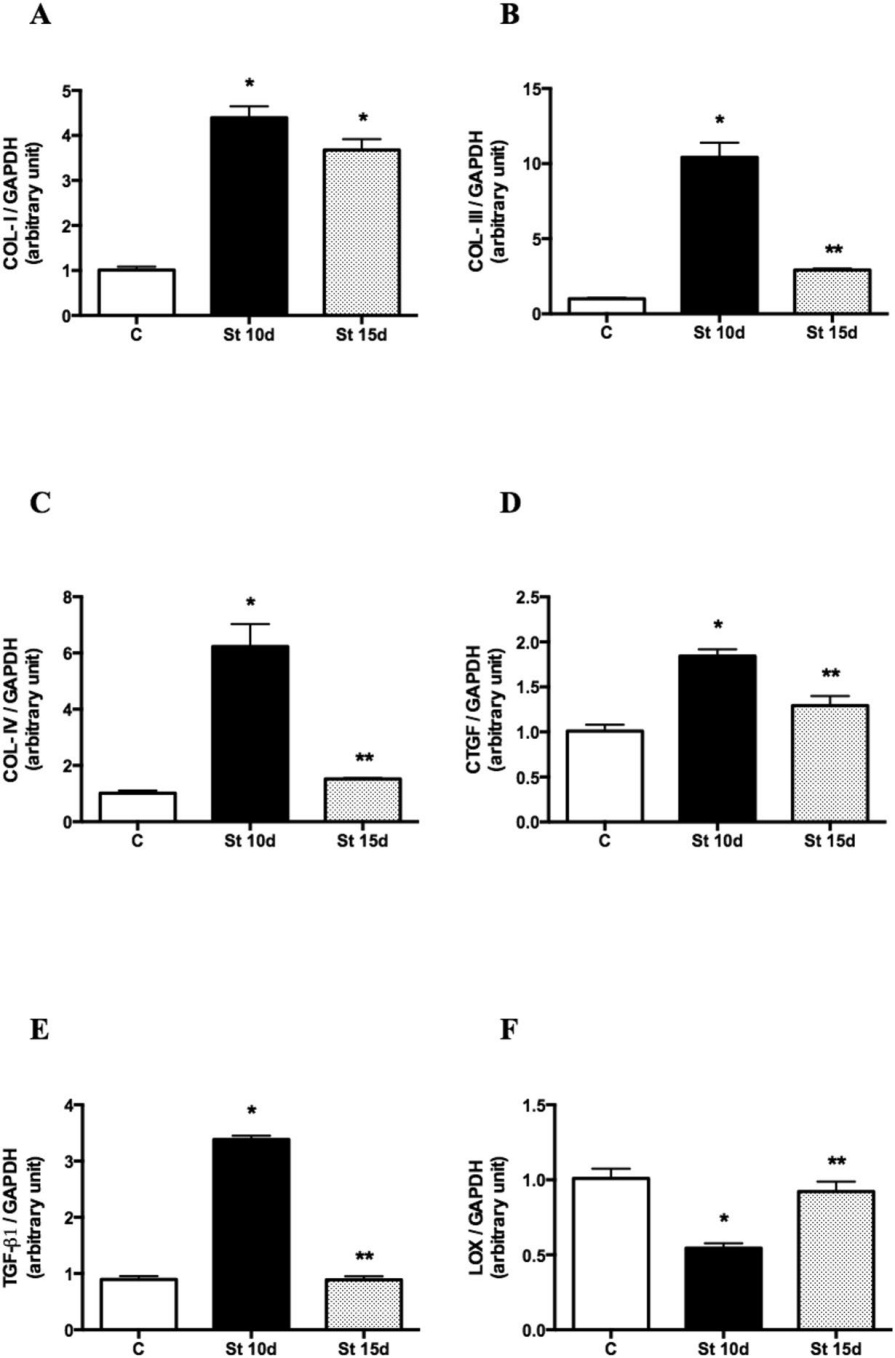
Gene expression by qPCR. Data are expressed as ratios of target genes COL-I (**A**), COL-III (**B**), COL-IV (**C**), CTGF (**D**), TGF-β (**E**) and LOX (**F**) to GAPDH in SOL muscle. Groups: control (**C**), 10 days of passive stretching (St 10 d) and 15 days of passive stretching (St 15d). Values are expressed as means ± SEM. One-way ANOVA, p < 0.05: * *vs*. C; ** *vs*. St 10d n = 6/group.

COL-I, III and IV (Fig. 3A–C; p < 0.0001), TGF-β1 and CTGF transcripts (Fig. 3D,E; p < 0.0001) increased significantly in the SOL muscles of the St10d group when compared to control rats. Lower COL-III, IV, TGF-β1 and CTGF mRNA levels were observed in the St15d group compared to St10d rats (Fig. 3B–E; p < 0.0001). LOX mRNA levels decreased in the St 10d group in relation to controls, whereas LOX transcripts were not statistically different from the control group, but higher in St 15d rats when compared to the St 10d group (Fig. 3F; p = 0.0002).

## Discussion

The present study demonstrated that 10 and 15 days of passive stretching mitigated COL-I content in the SOL muscles of rats, although MMP-2 activity declined after 10 sessions. Paradoxically, 10 days of passive stretching induced COL-I and COL-III synthesis, with concomitant upregulation of upstream signal transduction (CTGF and TGF-β1) at a transcriptional level. Despite the clear indication of collagen synthesis after 10 days of stretching, collagen cross-linking stabilization did not accompany this process, since low LOX mRNA levels were observed after 10 sessions of stretching. To the best of our knowledge, this is the first study that demonstrates the gene expression of ECM regulatory elements in response to passive stretching. Although results obtained in studies using denervation and immobilization are inconclusive, our findings indicated that longer periods of stretching alleviated collagen deposition in skeletal muscles, concomitant to different ECM regulator responses at a transcriptional level.

Sarcomerogenesis was observed after 15 days of passive stretching, suggesting that stretching frequency and duration may lead to muscle adaptations and parallel effects on ECM homeostasis (Fig. 1A). While one 40 min stretching session a week did not alter the serial sarcomere number in the SOL muscles of rats^35^, stretching every 3 days for 40 minutes increased the serial sarcomere number compared to the contralateral muscle^36^. In an earlier study, we found that denervated rat SOL muscles submitted to different periods of intermittent stretching showed no change in the serial sarcomere number^18^. In this study, 15 sessions of passive stretching induced sarcomerogenesis in SOL muscles. The total serial sarcomere number increased in SOL muscles stretched twice a week for 40 minutes, over eight consecutive weeks, which partially corroborates our findings^37^. It has been suggested that passive stretching could promote sarcomere addition if the muscle is lengthened enough to increase calcium release through activated calcium channels^38^.

Passive stretching alleviates muscle tissue stiffness^38^. As such, reduced muscle-tendon unit stiffness has been reported in response to 5 min of static stretching in human gastrocnemius muscles, suggesting changes in connective tissue properties^5,15^. In fact, passive resistance is influenced by deformation (lengthening) of the connective tissue (endomysium, perimysium, and epimysium) of the muscle belly in response to stretching^4^. Furthermore, a stretching protocol (ten 15 s repetitions with 30 s of rest between repetitions, applied post-immobilization for 10 consecutive days) led to the accumulation of connective interstitial tissue in the SOL muscles of female rats^33^. The authors suggested that connective tissue proliferation is a protective mechanism to prevent damage in the immobilized muscle. On the other hand, COL-I content declined in SOL muscles submitted to stretching over 10 and 15 days (Fig. 2), corroborating the findings of a previous study^34^. However, the stretching-induced molecular mechanisms involved in connective tissue homeostasis need to be better understood.

MMP-2 has been shown to play a role in the prolonged remodeling phase of skeletal muscle^23^. While MMP-2 activity decreased in the St 10 group in the present study, MMP-2 levels rose after 2, 4 and 7 days of lengthening contractions^39^. However, although a previous study showed no changes in MMP-2 and MMP-9 activity and gene expression in rat SOL muscles after a single stretching session^9^, four series of daily stretching raised MMP-2 activity in the tibialis anterior muscle^14^. In the present study, MMP-2 activity declined after 10 sessions of passive stretching, suggesting that the number of sessions is a key factor in modulating ECM turnover. The differences observed in the various studies may be due to the different methods used. Moreover, reduced MMP-2 activity may be related to the upregulation of collagen transcripts (I, III and IV), observed after 10 stretching sessions. In fact, 10 and 15 sessions of passive stretching reduced COL-I content (Fig. 2A), although intergroup COL-III and IV levels remained unchanged (Fig. 2B,C). This may be due to the involvement of other MMPs subtypes in COL-I degradation, including collagenases (MMP-1 and 13)^40,41^, as opposed to MMP-2 alone. Additionally, whereas COL-IV from the basal lamina of the muscle membrane has been identified as a target of MMP-9^42^, there were no changes in MMP-9 activity after 10 or 15 sessions of SOL muscle stretching (data not shown). Furthermore, MMP-9 was associated with activating latent TGF-β1 into a mature form, which induces fibrosis in dystrophic muscles^43^. On the other hand, MMP-9 gene expression rose in the SOL muscles of rats submitted to denervation followed by 10 stretching sessions^18^. Considering the differences between the studies, MMP expression seems to be more dependent on degeneration and regeneration processes in rat skeletal muscles^39^.

In the present study, 10 sessions of passive stretching prompted upregulation of COL-I and IV transcripts, while 15 sessions produced marked increases in COL-I and III mRNA levels in the SOL muscles of rats (Fig. 3A,B). In fact, in a previous study we demonstrated that short daily stretching sessions induced molecular reorganization of collagen fibrils after rat SOL muscle immobilization^44^. Lengthening contractions and/or mechanical deformation of extracellular connective tissues likely induces collagen synthesis in skeletal muscle, leading greater passive resistance when the muscle is stretched^4,39^. In another study, both COL-I content and TGF-β gene expression increased in denervated rat soleus muscles following 10 sessions of intermittent stretching on alternate days^18^. The mechanisms involved in these responses may be associated with pro-fibrotic signaling (CTGF and TGF-β1). Indeed, upregulation of CTGF and TGF-β1 mRNA levels was observed after 10 sessions of passive stretching, but there were no changes in these transcripts after 15 sessions (Fig. 3D,E). Mechanical stress seems to stimulate CTGF, which is involved in stretch-induced muscle fibrosis^45^. Repetitive stretch-relaxation cycles have been shown to induce the release of insulin-like growth factor (IGF-1) in differentiated muscle cells^46^, suggesting that “growth factors” are important targets related to ECM remodeling and skeletal muscle homeostasis^47,48^, and can also be regulated by stretching^18^. By contrast, *ex vivo* and *in vivo* stretching models resulted in higher TGF-β and type-1 procollagen levels in mouse tissues^48^.

Our findings regarding reduced COL-I deposition (Fig. 2A) may be associated with decreased LOX mRNA levels (Fig. 3F). On the other hand, mechanical loading (concentric, eccentric and isometric contractions) has been found to increase LOX transcripts in the tendons and gastrocnemius muscles of female rats^49^. However, with respect to skeletal muscle tissue, the effects of different stretching periods on LOX levels are not yet fully understood. Our study is the first to examine the effects of passive stretching on the genetic expression of LOX in rat muscles. Downregulation of LOX mRNA levels was observed after 10 passive stretching sessions, but LOX transcripts returned to basal values (control rats) after 15 sessions. LOX is thought to be important in the formation of lysine-derived cross-links between and within collagen molecules, contributing to stabilizing and strengthening collagen fibrils^28^. Thus, in contrast to the stretching-induced upregulation of molecules related to collagens synthesis (Fig. 3A–E), passive stretching seems to play a significant role in preventing covalent cross-links and stabilizing collagen fibrils in parallel with their intramuscular deposition (Fig. 3F). This was observed after 10 sessions of rat SOL muscle stretching, with no changes after 15 sessions. It is important to note that our findings do not support a mechanistic relationship between intramuscular collagen deposition and changes in the gene expression of factors related to collagen synthesis.

## Conclusions

In conclusion, passive stretching over 10 and 15 days mitigated COL-I deposition in the SOL muscle of rats. By contrast, 10 days of passive stretching induced COL-I and COL-III synthesis, with concomitant upregulation of upstream signal transduction (CTGF and TGF-β) at a transcriptional level. These responses may be associated with lower LOX mRNA levels in SOL muscles submitted to 10 sessions of passive stretching. Moreover, sarcomerogenesis was observed after 15 days of passive stretching, suggesting that stretching-induced muscle adaptations are time-dependent responses.

## Methods

### Experimental groups

Eighteen 3-month-old male Wistar rats were used (weight 350 ± 25 g). The animals were housed in plastic cages under controlled environmental conditions, with free access to water and standard food. The experimental procedures were approved by the University Ethics Committee (Number 25/2012) and in accordance with Guidelines for the Care and Use of Laboratory Animals. The rats were randomly divided into 3 groups of six animals and anesthetized by intraperitoneal injections of xylazine (12 mg.kg^−1^) and ketamine (95 mg.kg^−1^) to apply the stretching protocol. The animals were euthanized by an overdose of the anesthetic^8^.

### Stretching protocol

The stretching protocol was based on a previously validated method^8,9^. A single session of passive stretching (dorsi flexion) consisted of 10 sets of soleus (SOL) muscle stretching of the left ankle for 1 min, with a 30-second rest between sets. Rats underwent passive stretching for 10 (St 10d; n = 6) or 15 days (St 15d; n = 6). The SOL muscle has been widely used in our previous studies on skeletal muscle stretching^8,50^.

### Muscle Sample Collection

The animals were weighed at the end of the experimental period. The left SOL muscles were dissected and weighed 24 h after the last stretching session. Each SOL muscle was divided longitudinally into two segments: the lateral portion was used for the sarcomere measurements and the medial portion was divided into two parts (proximal and distal portions)^7,8,14^. The proximal portion was immediately frozen in isopentane pre-cooled in liquid nitrogen, stored at −80 °C and used to determine the morphology and cross sectional area (CSA), and in the immunofluorescence assay of collagen I, III and IV (COL-I, COL-III and COL-IV). The distal portion was divided into two equal segments, quickly frozen in liquid nitrogen, stored at −80 °C and used for zymography and mRNA analyses.

### Muscle CSA

Images of five different regions were acquired at 20x magnification, using a light microscope (Axiolab, Carl Zeiss, Jena, Germany) equipped with a digital camera (Sony DSC S75, Tokyo, Japan). In each image, the CSA of 70 random fibers was measured using Axiovision 4.7.1.0 software (Carl Zeiss, Jena, Germany), totaling 350 muscle fibers per animal (n = 6/group)^14^.

### Serial sarcomere number (sarcomerogenesis)

The sarcomere number within a single muscle fiber was determined as described by Williams and Goldspink^51^. The lateral portion of the SOL muscles was immersed in 2.5% glutaraldehyde (2.5%) for 3 h, transferred to a nitric oxide solution (30%) for two days and then stored in glycerol (50%). Five individual muscle fibers were teased out and fixed in gelatin-glycerin on a histological slide (n = 6/group)^52^.

Images were acquired at 20x magnification. The serial sarcomere number was determined using a light microscope (100x immersion objective; Axiolab, Carl Zeiss, Jena, Germany), equipped with a digital camera (Sony DSC S75, Tokyo, Japan). The sarcomere number was quantified with Axiovision 4.7.1.0 software (Carl Zeiss, Jena, Germany). The total serial sarcomere number was estimated based on the correlation between the number of sarcomeres identified in three 100 μm fields, totaling 300μm throughout the muscle fiber (n = 6/group)^53^.

### Zymography

Twenty-five milligrams of muscle was washed with cold saline and incubated in 2 mL of extraction buffer (10 mM cacodylic acid, pH 5.0, 150 mM NaCl, 1 μM ZnCl_2_, 20 mM CaCl_2_, 1.5 mM NaN_3_, and 0.01% Triton X-100) at 4 °C, with continuous mixing for 24 h. Protein concentration was determined using a BCA^TM^ protein assay kit (Pierce, Rockford, IL, USA), according to the manufacturer’s instruction.

Briefly, equal amounts of total protein (30 μg/ml) consisting of a pool of six animals per group (6 μg per animal) were subjected to electrophoresis in triplicate. Zymography gels consisted of 10% polyacrylamide impregnated with gelatin at a final concentration of 100 mg/ml H_2_O in the presence of sodium dodecyl sulfate (SDS), under nonreducing conditions. After 2 h of electrophoresis (100 V), the gels were washed twice for 20 min in a 2.5% Triton X-100 solution, and incubated at 37 °C for 20 h in a substrate buffer (50 mM Tris-HCl, pH 8.5, 5 mM CaCl_2_ and 0.02% NaN_3_). Next, the gels were stained with Coomassie brilliant blue R-250 for 30 min and then in methanol and acetic acid for 20 min. Gelatin-degrading enzymes were visualized as clear white bands against a blue background, indicating proteolysis of the substrate protein. The samples were also assayed in the presence of 15 mM EDTA, which inhibited MMP activity. The molecular mass of gelatinolytic activity was determined by comparison against a PageRulerTM Prestained Protein Ladder protein standard (Fermentas Life Sciences, Burlington, ON, Canada). Activity bands were identified as previously described, according to their molecular weights (72 kDa: Pro-MMP-2; 66 kDa: intermediary-MMP-2). MMP activity was quantified by densitometry analysis, using Gene Tools software, version 3.06 (Syngene, Cambridge, UK)^9,14,54^.

### RNA Isolation and Analysis

One frozen fragment of each muscle was homogenized and total RNA isolated using Trizol reagent (Invitrogen, Carlsbad, CA, USA), according to the manufacturer’s instructions. The extracted RNA was dissolved in tris-HCl and ethylene-diaminetetracetic acid (TE) (pH 7.6) and quantified spectrophotometrically. Purity was assessed by determining the absorbance ratio at 260 nm to that at 280 nm^55^. RNA integrity was then confirmed by inspection in 1% agarose gel stained with ethidium bromide (Invitrogen, Carlsbad, CA, USA), under ultraviolet light.

### Reverse Transcription

Reverse transcription of 1 µg of RNA was performed to synthesize cDNA. A reverse transcription (RT) reaction mixture containing 1 µg of cellular RNA, 5x reverse transcription buffer, a dNTP (Promega, Madison, WI) mixture with 0.2 mmol · L^−1^ each of dATP, dCTP, dGTP and 0.1 mol · L^−1^ of dTTP, 1 µl of oligo (dT) primer (Invitrogen, Carlsbad, CA, USA) and 200U of M-MLV RT enzyme (Promega, Madison, WI) was incubated at 70 °C for 10 min, 42 °C for 60 min, and then heated at 95 °C for 10 min before being quickly chilled on ice^19^.

### Oligonucleotide Primers

Oligonucleotide primers were designed for GAPDH (AF106860) and Collagen IV (alpha-1 type IV collagen - J04694) using Primer Express Software 2.0 (Applied Biosystems, Foster City, CA, USA). Oligonucleotide primers for COL1, COL3, TGFβ-1, CTGF, and LOX were described in accordance with another study^49^. The oligonucleotide primers are listed in Table 1.

**Table 1:** List of oligonucleotide primers.

	Forward	Reverse
COL-I	ATCAGCCCAAACCCCAAGGAGA	CGCAGGAAGGTCAGCTGGATAG
COL-III	TGATGGGATCCAATGAGGGAGA	GAGTCTCATGGCCTTGCGTGTTT
COL-IV	AGCGAGATGTTCAAGAAG	TGGACAGTGAGGTACACA
TGF-β1	CCCCTGGAAAGGGCTCAACAC	TCCAACCCAGGTCCTTCCTAAAGTC
CTGF	CAGGCTGGAGAAGCAGAGTCGT	CTGGTGCAGCCAGAAAGCTCAA
LOX	CAGGCACCGACCTGGATATGG	CGTACGTGGATGCCTGGATGTAGT
GAPDH	GATGCTGGTGCTGAGTATGTCG	GTGGTGCAGGATGCATTGCTGA

COL-I: type-I collagen; COL-III: type-III collagen; TGF-β1, transforming growth factor beta-1; CTGF: connective tissue growth factor; LOX: lysyl oxidase; GAPDH: Glyceraldehyde-3-Phosphate Dehydrogenase.

### Quantitative Polymerase Chain Reaction (qPCR) Analysis

Detection of mRNA for the different experimental and control samples was performed in a Rotor Gene 3000 instrument (Cobert’s, Sydney, Australia). The amplification mixes contained 1 μl of cDNA sample, 25 μl of SYBR green fluorescent dye, Master mix (Applied Biosystems, Foster City, CA, USA) and 180 nM of each primer, for a final volume of 50 μl. Thermal cycling conditions for COL-I, COL-III, COL-IV, TGF-β1, CTGF, LOX and GAPDH consisted of 10 min at 95 °C, 40 cycles of 15 s each at 94 °C and 30 s at 48 °C for COL-I, COL-III, COL-IV, TGF-β1, CTGF, and LOX and 56 °C for GAPDH, followed by 1 min at 72 °C and then 10 min at 72 °C. For each gene, all samples were amplified simultaneously in duplicate, in one assay run. Data were analyzed using the comparative cycle threshold (Ct) method. The target genes were normalized against GAPDH^8^. In addition, negative controls contained RNA and no M-MLV RT, thereby ensuring that the PCR product did not result from amplified genomic DNA. A blank was also performed, with no template sample and using only water, primers and SYBR green^56^.

### Immunofluorescence Analysis

Histological cross-sections (10 μm) of SOL muscles were fixed with 4% paraformaldehyde (Sigma P6148) in 0.2 M phosphate buffer (PB) for 10 min at room temperature, blocked with 0.1 M glycine in PB for 5 min, and permeabilized in 0.2% Triton X-100-PB for 10 min. Next, the sections were incubated in 1% bovine serum albumin (BSA) for 20 min at room temperature to block nonspecific binding, and incubated overnight with the primary antibody (diluted in 1% BSA) at 4 °C. Slides were washed with 0.1 MPB (3 times for 10 min each) and incubated with the secondary antibody (diluted in 1% BSA) for 2 h in a dark room. Slides were washed in 0.1 M PB (3 times for 10 min each) and mounted with Vectashield mounting medium containing 4,6-diamidino-2-phenylindole (catalog no. H-1200; Vector Laboratories)^18^. Negative control sections were not incubated with the primary antibody and experimental results were only considered if the controls showed no immunoreactivity. The primary antibodies used for immunostaining were monoclonal mouse anti-collagen I (1:100 dilution, catalog no. C2456; Sigma-Aldrich, St. Louis, MO, USA), monoclonal mouse anti-collagen III (1:100 dilution, catalog no. C7805; Sigma-Aldrich, St. Louis, MO, USA) and rabbit anti-collagen IV (1:200 dilution, catalog no. AB-19808; Abcam, Cambridge, MA, USA). The secondary antibody was Alexa Fluor 488 goat anti-mouse IgG, IgA, IgM (1:300 dilution, catalog no. A10667; Molecular Probes, Eugene, OR, USA); and rhodamine red goat anti-rabbit IgG (1:200 dilution, catalog no. Rb394; Molecular Probes, Eugene, OR, USA). For quantitative measurements of immunoreactivity (immunofluorescence), images were captured of five different regions in the middle belly of the SOL muscle (Axiocam, Carl Zeiss, Jena, Germany) at 20x magnification^57^. The images were quantified using ImageJ analysis software (NIH, Bethesda, MD, USA) and expressed as percentage (%) of total area^18,56^.

### Statistical analysis

Levene’s test was applied to evaluate the homogeneity of the results. Results are expressed as means ± SEM. Parametric tests were used because the data were normally distributed (Shapiro-Wilk test) with homogeneous variances. Statistical analysis was performed by one-way analysis of variance (ANOVA), followed by the Bonferroni post hoc test. Significance was set at 5% (p ≤ 0.05).

## References

[1] Williams PE (1988). Effect of intermittent stretch on immobilised muscle. Ann. Rheum. Dis..

[2] Williams PE (1990). Use of intermittent stretch in the prevention of serial sarcomere loss in immobilised muscle. Ann. Rheum. Dis..

[3] Ikeda S, Yoshida A, Matayoshi S, Horinouchi K, Tanaka N (2004). Induction of Myogenin Messenger Ribonucleic Acid in Rat Skeletal Muscle after 1 Hour of Passive Repetitive Stretching. Arch. Phys. Med. Rehabil..

[4] Gajdosik RL (2001). Passive extensibility of skeletal muscle: Review of the literature with clinical implications. Clinical Biomechanics.

[5] Nakamura M, Ikezoe T, Takeno Y, Ichihashi N (2011). Acute and prolonged effect of static stretching on the passive stiffness of the human gastrocnemius muscle tendon unit *in vivo*. J. Orthop. Res..

[6] Kamikawa, Y., Ikeda, S., Harada, K., Ohwatashi, A. & Yoshida, A. Passive repetitive stretching for a short duration within a week increases myogenic regulatory factors and myosin heavy chain mRNA in rats’ skeletal muscles. *Sci*. *World J*. **2013** (2013). 10.1155/2013/493656 PMC3676914 23766692

[7] Gomes AR (2006). The effect of 30 minutes of passive stretch of the rat soleus muscle on the myogenic differentiation, myostatin, and atrogin-1 gene expressions. Arch. Phys. Med. Rehabil..

[8] Peviani SM, Gomes ARS, Moreira RFC, Moriscot AS, Salvini TF (2007). Short bouts of stretching increase myo-D, myostatin and atrogin-1 in rat soleus muscle. Muscle and Nerve.

[9] Peviani SM, Gomes ARS, De Araujo HSS, Salvini TF (2009). MMP-2 is not altered by stretching in skeletal muscle. Int. J. Sports Med..

[10] Loughna P, Goldspink G, Goldspink DF (1986). Effect of inactivity and passive stretch on protein turnover in phasic and postural rat muscles. J. Appl. Physiol..

[11] Goldspink, G., Williams, P. & Simpson, H. Gene expression in response to muscle stretch. *Clin*. *Orthop*. *Relat*. *Res*. S146–S152, 10.1097/01.blo.0000031972.69509.68 (2002). 10.1097/00003086-200210001-00017 12394463

[12] Freitas SR, Mil-Homens P (2015). Effect of 8-Week High-Intensity Stretching Training on Biceps Femoris Architecture. J. Strength Cond. Res..

[13] Zöllner, A. M., Abilez, O. J., Böl, M. & Kuhl, E. Stretching Skeletal Muscle: Chronic Muscle Lengthening through Sarcomerogenesis. *Plos One* **7** (2012). 10.1371/journal.pone.0045661 PMC3462200 23049683

[14] Peviani SM (2010). Stretching and electrical stimulation regulate the metalloproteinase-2 in rat denervated skeletal muscle. Neurol. Res..

[15] Kubo K, Kanehisa H, Fukunaga T (2002). Effects of transient muscle contractions and stretching on the tendon structures *in vivo*. Acta Physiol. Scand..

[16] Cação-Benedini LO, Ribeiro PG, Prado CM, Chesca DL, Mattiello-Sverzut AC (2014). Immobilization and therapeutic passive stretching generate thickening and increase the expression of laminin and dystrophin in skeletal muscle. Brazilian J. Med. Biol. Res..

[17] Cornachione, A. S., Cação-Benedini, L. O., Benedini-Elias, P. C. O., Martinez, E. Z. & Mattiello-Sverzut, A. C. Effects of 40 min of maintained stretch on the soleus and plantaris muscles of rats applied for different periods of time after hindlimb immobilization. *Acta Histochem*., 10.1016/j.acthis.2012.11.008 (2013). 10.1016/j.acthis.2012.11.008 23287280

[18] Faturi FM (2016). Intermittent stretching induces fibrosis in denervated rat muscle. Muscle and Nerve.

[19] Russo TL (2010). Stretching and electrical stimulation reduce the accumulation of MyoD, myostatin and atrogin-1 in denervated rat skeletal muscle. J. Muscle Res. Cell Motil..

[20] Gillies AR, Lieber B, Lieber RL (2012). Structure and Function of the Skeletal Muscle Extracellular Matrix. Muscle Nerve.

[21] Kragstrup TW, Kjaer M, Mackey AL (2011). Structural, biochemical, cellular, and functional changes in skeletal muscle extracellular matrix with aging. Scandinavian Journal of Medicine and Science in Sports.

[22] Alameddine HS, Morgan JE (2016). Matrix Metalloproteinases and Tissue Inhibitor of Metalloproteinases in Inflammation and Fibrosis of Skeletal Muscles. J. Neuromuscul. Dis..

[23] Kherif S (1999). Expression of matrix metalloproteinases 2 and 9 in regenerating skeletal muscle: a study in experimentally injured and mdx muscles. Dev. Biol..

[24] Verrecchia F, Chu ML, Mauviel A (2001). Identification of Novel TGF-β/Smad Gene Targets in Dermal Fibroblasts using a Combined cDNA Microarray/Promoter Transactivation Approach. J. Biol. Chem..

[25] Leask A, Holmes A, Abraham DJ (2002). Connective tissue growth factor: a new and important player in the pathogenesis of fibrosis. Curr. Rheumatol. Rep..

[26] Leask A, Abraham DJ (2004). TGF-beta signaling and the fibrotic response. FASEB J..

[27] Mendias CL (2012). Transforming growth factor-beta induces skeletal muscle atrophy and fibrosis through the induction of atrogin-1 and scleraxis. Muscle and Nerve.

[28] Kagan HM, Li W (2003). Lysyl oxidase: Properties, specificity, and biological roles inside and outside of the cell. J. Cell. Biochem..

[29] Willems MET, Miller GR, Stauber WT (2001). Force deficits after stretches of activated rat muscle-tendon complex with reduced collagen cross-linking. Eur. J. Appl. Physiol..

[30] Karpakka J, Vaananen K, Orava S, Takala TE (1990). The effects of preimmobilization training and immobilization on collagen synthesis in rat skeletal muscle. Int. J. Sports Med..

[31] Järvinen TAH, Józsa L, Kannus P, Järvinen TLN, Järvinen M (2002). Organization and distribution of intramuscular connective tissue in normal and immobilized skeletal muscles. J. Muscle Res. Cell Motil..

[32] Williams PE, Catanese T, Lucey EG, Goldspink G (1988). The importance of stretch and contractile activity in the prevention of connective tissue accumulation in muscle. J. Anat..

[33] Mattiello-Sverzut AC (2006). Morphological effects of electrical stimulation and intermittent muscle stretch after immobilization in soleus muscle. Histol. Histopathol..

[34] Zotz TG (2016). Acute effects of stretching exercise on the soleus muscle of female aged rats. Acta Histochem..

[35] Gomes ARS, Coutinho EL, França CN, Polonio J, Salvini TF (2004). Effect of one stretch a week applied to the immobilized soleus muscle on rat muscle fiber morphology. Brazilian J. Med. Biol. Res..

[36] Coutinho EL, Gomes ARS, França CN, Oishi J, Salvini TF (2004). Effect of passive stretching on the immobilized soleus muscle fiber morphology. Brazilian J. Med. Biol. Res..

[37] Secchi KV, Morais CP, Cimatti PF, Tokars E, Gomes ARS (2008). Effects of stretching and resistive exercise in rat skeletal muscle. Rev. Bras. Fisioter..

[38] Riley DA, Van Dyke JM (2012). The Effects of Active and Passive Stretching on Muscle Length. Phys. Med. Rehabil. Clin. N. Am..

[39] Koskinen SOA (2002). Short-term effects of forced eccentric contractions on collagen synthesis and degradation in rat skeletal muscle. Pflugers Arch..

[40] Singh A, Nelson-Moon ZL, Thomas GJ, Hunt NP, Lewis MP (2000). Identification of matrix metalloproteinases and their tissue inhibitors type 1 and 2 in human masseter muscle. Arch. Oral Biol..

[41] Wu N, Jansen ED, Davidson JM (2003). Comparison of Mouse Matrix Metalloproteinase 13 Expression in Free-Electron Laser and Scalpel Incisions during Wound Healing. J. Invest. Dermatol..

[42] Li H (2009). Tumor Necrosis Factor-related Weak Inducer of Apoptosis Augments Matrix Metalloproteinase 9 (MMP-9) Production in Skeletal Muscle through the Activation of Nuclear Factor-κB-inducing Kinase and p38 Mitogen-activated Protein Kinase. J. Biol. Chem..

[43] Li H, Mittal A, Makonchuk DY, Bhatnagar S, Kumar A (2009). Matrix metalloproteinase-9 inhibition ameliorates pathogenesis and improves skeletal muscle regeneration in muscular dystrophy. Hum. Mol. Genet..

[44] Coutinho EL, DeLuca C, Salvini TF, Vidal BC (2006). Bouts of passive stretching after immobilization of the rat soleus muscle increase collagen macromolecular organization and muscle fiber area. Connect. Tissue Res..

[45] Schild C, Trueb B (2002). Mechanical Stress Is Required for High-Level Expression of Connective Tissue Growth Factor. Exp. Cell Res..

[46] Perrone CE, Fenwick-Smith D, Vandenburgh HH (1995). Collagen and stretch modulate autocrine secretion of insulin-like growth factor-1 and insulin-like growth factor binding proteins from differentiated skeletal muscle cells. J. Biol. Chem..

[47] Burks TN, Cohn RD (2011). Role of TGF-β signaling in inherited and acquired myopathies. Skelet. Muscle.

[48] Bouffard NA (2008). Tissue stretch decreases soluble TGF-β1 and type-1 procollagen in mouse subcutaneous connective tissue: Evidence from *ex vivo* and *in vivo* models. J. Cell. Physiol..

[49] Heinemeier KM (2007). Expression of collagen and related growth factors in rat tendon and skeletal muscle in response to specific contraction types. J. Physiol..

[50] Koskinen SO (2001). Acute exercise induced changes in rat skeletal muscle mRNAs and proteins regulating type IV collagen content. Am. J. Physiol. Regul. Integr. Comp. Physiol..

[51] Williams PE, Goldspink G (1971). Longitudinal Growth of Striated Muscle Fibres. J. Cell Sci..

[52] Goldspink G, Goldspink G (1968). Sarcomere Length during Post-Natal Growth of Mammalian Muscle Fibres. J. Cell Sci..

[53] Williams PE, Goldspink G (1978). Changes in sarcomere length and physiological properties in immobilized muscle. J. Anat..

[54] Guzzoni V (2017). Reduced collagen accumulation and augmented MMP-2 activity in left ventricle of old rats submitted to high-intensity resistance training. J. Appl. Physiol..

[55] Durigan JLQ (2014). Neuromuscular electrical stimulation alters gene expression and delays quadriceps muscle atrophy of rats after anterior cruciate ligament transection. Muscle Nerve.

[56] Durigan JLQ (2014). Neuromuscular Electrical Stimulation Induces Beneficial Adaptations in the Extracellular Matrix of Quadriceps Muscle After Anterior Cruciate Ligament Transection of Rats. Am. J. Phys. Med. Rehabil..

[57] Vieira Ramos, G. *et al*. Cryotherapy reduces inflammatory response without altering muscle regeneration process and extracellular matrix remodeling of rat muscle. *Sci*. *Rep*. **6** (2016). 10.1038/srep18525 PMC4698758 26725948

